# Editorial: The Vestibular System in Cognitive and Memory Processes in Mammalians

**DOI:** 10.3389/fnint.2015.00055

**Published:** 2015-11-10

**Authors:** Stéphane Besnard, Christophe Lopez, Thomas Brandt, Pierre Denise, Paul F. Smith

**Affiliations:** ^1^COMETE, Institut National de la Santé et de la Recherche Médicale U1075, Normandy UniversityCaen, France; ^2^Centre National de la Recherche Scientifique, NIA UMR 7260, Aix Marseille UniversitéMarseille, France; ^3^German Center for Vertigo and Balance DisordersMunich, Germany; ^4^Department of Pharmacology and Toxicology and the Brain Health Research Centre, University of Otago Medical SchoolDunedin, New Zealand

**Keywords:** sensory organ, vertigo, dementia, cognition, perception, balance disorders

In the nineteenth century Pierre–Jean–Marie Flourens (1825) and Ernst Mach described the vestibular system and its peripheral organs while Robert Barany, rewarded by the Nobel prize in 1914, was the first to investigate vestibular disorders with caloric tests making surgical treatments of the vestibular organ possible. Recently, Graf and Klam (2006) have reminded us that this ancient sensory system appeared more than 500 million years ago. Logically its influence would most likely not be restricted to balance reflexes at the brainstem level; it must have also shaped our brain. The vestibular system is the one sensory organ dedicated to gravity perception, which along with light and oxygen served as a motor of evolution. In the 1950s the groups of Otto–Joachim Grüsser in Germany, Wilder Penfield in Canada, and later the group of Alain Berthoz in France, demonstrated in elegant experiments on awake monkey (Guldin and Grüsser, 1998), epileptic patient (Penfield, 1957), and neurologically-normal human (Lobel et al., 1999) the existence of vestibular projections to the cortex and how they combine with visual and proprioceptive information. An increasing number of researchers, often fervent disciples, have built on these findings to produce a spate of publications that have consolidated the evidence for a sense of verticality and three-dimensional body representations within the vestibular cortical areas. In the 1990s Paul Smith and colleagues examined vestibular processing in the hippocampus and its role in spatial memory. Exploring this topic in the rodent (Smith, 1997), they began to elucidate the secrets and the previously silent functions of the vestibular system. These findings led to increasing clarity about how vestibular degeneration may be related to some aspects of dementia (Previc, 2013), psychiatric diseases (Gurvich et al., 2013), and cognitive impairments in the elderly (Bigelow et al., 2015; Semenov et al., 2015). The research by Marianne Dieterich and Thomas Brandt has examined the bilateral organization of multiple multisensory cortical areas and revealed the vestibular dominance of the non-dominant hemisphere (Dieterich et al., 2003). They addressed the following questions: how is one global percept of motion and orientation in space formed, and does this dominance determine the lateralization of brain function such as handedness (Brandt and Dieterich, 2015)? A vestibular contribution to the most crucial aspects of the human sense of self and self-consciousness has recently been highlighted by neurological and neuroscientific investigations: vestibular signals contribute to the experience that the self is located within the boundaries of the body (Blanke et al., 2004; Lopez et al., 2008) and may even be involved in self-other discrimination and interactions (Lenggenhager and Lopez, 2015).

In this *Frontiers in Integrative Neuroscience* Research Topic initiated by Sidney Simon, twenty-four articles highlight recent discoveries in the field of vestibular cognition, including: (1) Anatomy of the vestibulo-cortical pathways; (2) Spatial navigation and memory; (3) Spatial cognition, bodily and self-motion perception; (4) Vestibular stimulation and rehabilitation; (5) Posture and motor control; (6) Vestibular disorders and compensation; and (7) Development of vestibular function.

## Anatomy of the vestibulo-cortical pathways

A better understanding of the vestibulo-thalamo-cortical pathways and cortical vestibular processing is needed to fully understand the reciprocal interactions between vestibular processing and cognition. Hitier et al. (2014) provide a comprehensive review of the pathways running from the vestibular apparatus to the cortex, with a focus on the vestibulo-hippocampal pathways. Ventre-Dominey (2014) proposes that two separate cortical vestibular subsystems underpin velocity and inertia processing. A better definition of the vestibular cortex is provided on the basis of clinical and neuroimaging investigations in brain-damaged patients by Brandt et al. (2014), and on the basis of electrophysiological investigations in epileptic patients by Hewett and Bartolomei (2013).

## Spatial navigation and memory

Yoder and Taube (2014) and Smith and Zheng (2013) offer a recent overview of the vestibular contribution to spatial learning and navigation through the head direction and place cells and how vestibular information is integrated into higher navigation centers. Jacob et al. (2014) provide a review and original data relating to the role of the entorhinal cortex (EC) in spatial memory. Despite the evidence that place cells in the hippocampus are adversely affected by the loss of vestibular function, there have been no data reported relating to grid cells in the EC. Here the authors present evidence that inactivation of the vestibular system in rats using tetrodotoxin injections results in a decrease in power in velocity-related theta EEG (5–12 Hz) in the EC, suggesting a disruption of grid cell activity. Cullen (2014) reviews the recent evidence that vestibular signals that are transmitted to higher centers of the brain concerned with spatial memory are in fact multi-modal and more complex than previously thought. She discusses the implications of these findings for the analysis of active and passive movements in relation to spatial memory. Interestingly, Previc et al. (2014) point out how vestibular degeneration may contribute to spatial memory impairments and more generally raise the question of the consequences of progressive sensory loss in the elderly.

## Spatial cognition, bodily, and self-motion perception

On the basis of recent evidence that vestibular processing is involved in changing the visuo-spatial perspective and in self-other discrimination, Deroualle and Lopez (2014) propose a vestibular contribution to several sensorimotor mechanisms that are important for social cognition. Psychological investigations have recently demonstrated how vestibular information may play a role in spatial cognition like mental imagery, bodily self-consciousness and self-motion perception, including its influences on emotional aspects and mood as reported by Mast et al. (2014), Pfeiffer et al. (2014), and Tremblay et al. (2013), with an overview of the higher cognitive processes of self-consciousness. Mast et al. (2014) also reports how vestibular disorders and some psychiatric symptoms may be entangled, completing the review of Gurvich et al. (2013).

## Vestibular stimulation and neurological rehabilitation

Caloric (CVS) and galvanic (GVS) vestibular stimulation have long been used to stimulate the vestibular receptors in neurologically normal participants and have been used to improve recovery in stroke patients. Palla and Lenggenhager (2014) review the importance of these methods for studying cognition and Bottini et al. (2013) highlight the relevance of CVS for studying somatosensory perception and bodily awareness. Two clinical applications of CVS and GVS are proposed by Wilkinson and colleagues. Wilkinson et al. (2013) present a series of case reports on the effects of CVS on post-stroke aphasia. They report that daily CVS for four consecutive weeks was associated with a significant improvement in picture and responsive naming, immediately and at a 1 week follow-up, suggesting that CVS may reduce aphasic symptoms. The same group (Wilkinson et al., 2014) also demonstrates that subliminal GVS can improve hemispatial neglect in right hemisphere stroke patients up to 1 month after the application of GVS, providing evidence for long-term therapeutic effects of GVS. Mast et al. (2014) also report beneficial effects of vestibular stimulation on psychiatric disorders, expanding the field of the vestibular sensory therapy.

## Posture and motor control

Lacquaniti et al. (2013) review neuroimaging data that have recently revealed the role of the temporo-parietal junction, insula, and retroinsular cortex in estimating the consequences of gravity for visual perception and motor preparation. In an original research article, Ferrè et al. (2013) describe the influence of GVS on the generation of motor sequences. They show that right cathodal GVS increases the generation of novel motor sequences. Bernard-Demanze et al. (2014) report original research on static and dynamic postural stability in cochlear implant (CI) patients. They demonstrate that, compared to controls, the CI patients exhibit increased postural instability, especially with the eyes closed, and that these deficits persist during dual tasking with visual or auditory memory tasks.

## Vestibular disorders and compensation

Lopez (2013) reviews the consequences of vestibular disorders for bodily perceptions, the sense of self and self-consciousness. Peripheral vestibular disorders may alter the perceived shape and size of the body, alter the experience of owning the body and evoke the experience that the self is strange, unreal and disembodied. Tighilet et al. (2014) describe the consequences of vestibular deafferentation for the cat histaminergic system and show that plasticity of the histamine H_3_ receptors supports vestibular compensation.

## Development of vestibular functions

Wiener-Vacher et al. (2013) review the evidence for the importance of vestibular information for hippocampal representations of space and consider the implications of this for the development of spatial navigation and orientation in children, a subject which has been relatively neglected. They hypothesize that the loss of vestibular function before critical stages of development will lead to specific cognitive deficits in humans. Jamon (2014) confirms the existence of critical periods for the adaptation to gravity from rodent models exposed to micro- and hyper-gravity environments and that vestibular graviceptors are calibrated from gravity experience after birth.

Future studies on the vestibular system will certainly continue to bring new neurophysiological and neuropathological findings as well as innovative concepts. Ideas are emerging on the cerebral benefits of vestibular stimulation which might even spread beyond the brain. For example, recent studies report that the vestibular organ can influence bone and muscle remodeling (Vignaux et al., 2015), help synchronize circadian rhythms (Fuller et al., 2002; Martin et al., 2015), and regulate vegetative and postural blood pressure (Normand et al., 1997; Yates et al., 2014).

The sky's the limit for new ideas and developments in vestibular therapy (both pharmacological molecules and physical devices). One only has to consider the limited range of pharmaceuticals now available for treating vertigo and motion sickness to realize the great demand for new strategies. Such ideas include a highly optimized GVS, a centrifuge creating g levels for long-term space missions, GVS for modulating motor hemi-neglect, vestibular compensation, and more generally motor and balance disorders (Rizzo-Sierra et al., 2014; An, 2015), as well as multisensory “virtual” rehabilitation suggested by recent advances in immersive virtual environments.

This *Frontiers in Integrative Neuroscience* Research Topic has given us the opportunity to gather and share the latest findings on cognitive and memory processes related to the vestibular system. Twenty-four articles and at least ninety-four authors, whom we sincerely thank, have contributed to the success of this Research Topic. They have convincingly demonstrated the growing popularity of the vestibular sense organ in the scientific community and pointed to promising topics of future research!

## Funding

A Part of the research's leading these results has received fundings from the Centre National d'Etude Spatiale (CNES), The Basse-Normandie Region, The European Union's Seventh Framework Programme FP7/2007-2013, the People Programme (Marie Curie Actions) of the European Union's Seventh Framework Programme (FP7/2007-2013) under REA grant agreement number 333607 to CL (“BODILYSELF, vestibular and multisensory investigations of bodily self-consciousness,” The Marsden Fund (Royal Society of New Zealand), The Health Research Council of New Zealand, The New Zealand Neurological Foundation, German Federal Ministry of Education and Research, and The Hertie-Foundation.

### Conflict of interest statement

The authors declare that the research was conducted in the absence of any commercial or financial relationships that could be construed as a potential conflict of interest.
